# Structural and Evolutionary Relationships of Melanin Cascade Proteins in Cnidarian Innate Immunity

**DOI:** 10.1093/icb/icae115

**Published:** 2024-07-18

**Authors:** Emily W Van Buren, Ivan E Ponce, Kelsey M Beavers, Alexia Stokes, Mariah N Cornelio, Madison Emery, Laura D Mydlarz

**Affiliations:** Department of Biology, University of Texas at Arlington, Arlington, TX 76019, USA; Department of Biology, University of Texas at Arlington, Arlington, TX 76019, USA; Department of Biology, University of Texas at Arlington, Arlington, TX 76019, USA; Texas Advanced Computing Center, University of Texas at Austin, Austin, TX 78758, USA; Department of Biology, University of Texas at Arlington, Arlington, TX 76019, USA; Department of Biology, University of Texas at Arlington, Arlington, TX 76019, USA; Department of Biology, University of Texas at Arlington, Arlington, TX 76019, USA; Department of Biology, University of Texas at Arlington, Arlington, TX 76019, USA

## Abstract

Melanin is an essential product that plays an important role in innate immunity in a variety of organisms across the animal kingdom. Melanin synthesis is performed by many organisms using the tyrosine metabolism pathway, a general pathway that utilizes a type-three copper oxidase protein, called PO-candidates (phenoloxidase candidates). While melanin synthesis is well-characterized in organisms like arthropods and humans, it is not as well-understood in non-model organisms such as cnidarians. With the rising anthropomorphic climate change influence on marine ecosystems, cnidarians, specifically corals, are under an increased threat of bleaching and disease. Understanding innate immune pathways, such as melanin synthesis, is vital for gaining insights into how corals may be able to fight these threats. In this study, we use comparative bioinformatic approaches to provide a comprehensive analysis of genes involved in tyrosine-mediated melanin synthesis in cnidarians. Eighteen PO-candidates representing five phyla were studied to identify their evolutionary relationship. Cnidarian species were most similar to chordates due to domain presents in the amino acid sequences. From there, functionally conserved domains in coral proteins were identified in a coral disease dataset. Five stony corals exposed to stony coral tissue loss disease were leveraged to identify 18 putative tyrosine metabolism genes, genes with functionally conserved domains to their *Homo sapiens* counterpart. To put this pathway in the context of coral health, putative genes were correlated to melanin concentration from tissues of stony coral species in the disease exposure dataset. In this study, tyrosinase was identified in stony corals as correlated to melanin concentrations and likely plays a key role in immunity as a resistance trait. In addition, stony coral genes were assigned to all modules within the tyrosine metabolism pathway, indicating an evolutionary conservation of this pathway across phyla. Overall, this study provides a comprehensive analysis of the genes involved in tyrosine-mediated melanin synthesis in cnidarians.

## Introduction

Melanin is a highly conserved multifunctional pigment best known for roles in mammalian skin and hair pigmentation (D'Mello et al. 2016). In invertebrates, melanin has many other functions, including those related to innate immunity (True 2003; Christensen et al. 2005; Liu et al. 2016; Qiao et al. 2016; Whitten and Coates 2017; Ehrlich and Zuk 2019). For example, insects utilize melanin in embryonic development, coloration, and pathogen encapsulation (Dimopoulos 2003; Okada et al. 2006; Zou et al. 2008; Córdoba-Aguilar et al. 2009; Lee et al. 2018). In ecologically important cnidarians, such a stony corals, melanin production is associated with wound healing and disease, but the mechanisms by which these organisms produce these phenotypes are unresolved (Zhuang et al. 2009; Fuess et al. 2018; Bailey et al. 2019; Ricci et al. 2019; Aziz et al. 2021). An understanding of the melanin cascade in basal metazoans, such as cnidarians, is important as it will provide better insight into the evolution of these pathways across phyla and will help us better understand cnidarian's stress and disease responses.

The pathways of melanin synthesis such as melanogenesis and tyrosine metabolism are well characterized in organisms like *Homo sapiens* and *Drosophila melanogaster*. The core components of these melanin synthesis pathways include upstream cell signaling pathways such as Wnt or MAPK, the transcription factor MITF (Melanocyte Inducing Transcription Factor or Microphtalmia-Associated Transcription Factor), and rate limiting phenoloxidase (PO) enzymes. Melanin synthesis is initiated by the presence of tyrosine, indicating a conserved tyrosine-mediated melanin synthesis process (Nappi and Christensen 2005; D'Mello et al. 2016; Bailey et al. 2019; Koike and Yamasaki 2020; Yu et al. 2021). A variety of studies in several species indicate that in melanin synthesis, the rate limiting enzymatic reactions and non-enzymatic reactions are equally important in creating the melanin product. The rate limiting enzymes perform the catalyzation and hydroxylation of monophenols into diphenols and quinone intermediates through an enzymatic process (Hoeger and Harris 2020). Accordingly, non-enzymatic reactions in quinone-radical intermediates create the melanin product. There are a variety of enzymes that can catalyze the rate-limiting step of melanin synthesis. While different protein families make up these rate limiting enzymes, all are capable of producing melanin.

Due to the diversity in protein families that can perform the rate-limiting step across phyla, we broadly categorize these enzymes as PO-candidates, proteins that are either PO or PO-like (Hoeger and Harris 2020; Pavan et al. 2020). PO-candidates are all multicopper oxidase proteins that can have two to six copper atoms at their active sites. Hemocyanins, tyrosinases (TYRs), and catecholoxidases are type-three copper proteins enzymes that perform this rate limiting step and only have two copper atoms at their active site. Laccases are a general multicopper oxidase that perform the melanin rate-limiting step and have more than two copper atoms at the active site (Janusz et al. 2020; Pavan et al. 2020). Laccase PO-candidates are annotated in several pathways that produce a melanin product, including melanogenesis and generic annotations of melanin synthesis pathways in literature (D'Mello et al. 2016; Liu et al. 2016; Whitten and Coates 2017).

Studies in *H. sapiens* and arthropods have driven the progress in melanin research as they have played important roles in life stages, coloration, and immunity. In these organisms, the variety of PO-candidates are due to the roles they play in that organism. For examples, humans have PO-candidates TYR and laccase that are used for radiation defense and inflammation (Gasque and Jaffar-Bandjee 2015; Koike and Yamasaki 2020). However, insects have a variety of prophenoloxidases PO-candidates characterized by their hemocyanin domains, which encapsulate different types of pathogens, including bacteria, fungi, and viruses (Christensen et al. 2005; Wang et al. 2017, 2018). Arthropods have the capability of expressing all PO-candidates, including hemocyanin, TYR-like and laccase-like, and have been reported the expression of a variety of these PO-candidates during pathogen or stress challenges (Aladaileh et al. 2007; Yu et al. 2014; Tassanakajon et al. 2018). The *H. sapiens* and the arthropod pathway differ in the types and uses of PO-candidates at the rate limiting steps. Building of the information of PO-candidates in model organisms, the understanding of melanin synthesis has expanded in non-model organisms through comparative immunology approaches on model to non-model organisms (Esposito et al. 2012; Bailey et al. 2019).

Cnidarians synthesize and use melanin as a part of their innate immune system (Anctil 2009; Zaragoza et al. 2014; Parisi et al. 2020). Melanin production occurs as a reaction to stress, wound healing, pathogen or pathogen associated molecular patterns (PAMPs) exposure, and coral bleaching (Palmer et al. 2010; Mydlarz and Palmer 2011; Palmer et al. 2011; Palmer et al. 2011; van de Water et al. 2015; Fuess et al. 2018; Palmer and Baird 2018; Bailey et al. 2019; Ricci et al. 2019; Parisi et al. 2020). Melanin can appear as a visible phenotype, which has been seen in the purpling of sea fans and black spot development on Eunicea during disease events (Mydlarz and Harvell 2007; Fuess et al. 2018; Ricci et al. 2019). Gene expression studies provide further support of PO-candidates in the coral pathogen response, with differential expression of some PO-candidates in disease exposure, high expression of PO-candidates being linked to disease resistance, and TYR being characteristic of immune cell types (Mydlarz and Palmer 2011; Vidal-Dupiol et al. 2014; van de Water et al. 2015; Connelly et al. 2020; Levy et al. 2021; MacKnight et al. 2022). Understanding an immune pathway in cnidarians is vital now, as coral disease is a rising threat to reefs. Coral disease poses an existential threat to Caribbean coral reefs, as seen in the stony corals afflicted by stony coral tissue loss disease (SCTLD), which has resulted in high mortality rates and loss over overall coral coverage on reefs (Alvarez-Filip et al. 2022).

In this study, PO-candidates and melanin synthesis cascades in cnidarians were surveyed using phylogenetic protein family comparisons and annotation of specific genes in the pathways. This study had two main goals: (i) to put the coral melanin pathway in comparative evolutionary context using protein sequence data and bioinformatic tools, and (ii) to use an existing dataset with active disease (SCTLD) to identify melanin synthesis and PO-candidates that may be important to coral's ability to survive disease. To accomplish the first goal, protein sequences of PO-candidates from a variety of species were compared using sequence alignment software to elucidate the evolutionary relationship in this protein family. In the second goal, the orthologous transcriptomic dataset from the active disease SCTLD study was leveraged to understand the connection between melanin synthesis and PO-candidate genes. This study quantified melanin concentration in samples from this active disease dataset to correlate to the putative orthogroups that represent a melanin synthesis pathway. Since there is not one universal gene responsible for melanin synthesis and the pathway is not understood in cnidarians, utilizing this study that encompasses five stony coral species provides insight into evolution of innate immunity and processes important to coral's ability to survive disease.

## Materials and methods

### Annotation of melanin production pathways

Melanin synthesis encompasses multiple pathways across a variety of species. The two major pathways annotated on the Kyoto Encyclopedia of Genes and Genomes (KEGG) (Kanehisa and Goto 2000; Kanehisa 2019; Kanehisa et al. 2021) are melanogenesis [KEGG:04916] and tyrosine metabolism [KEGG:00350]. The generalized tyrosine metabolism pathway encompasses a wide variety of enzymes that perform melanin synthesis regardless of specialization within species (Vavricka et al. 2014), while melanogenesis is exclusive to chordates with melanosomes (D'Mello et al. 2016), so tyrosine metabolism was chosen for this study. For the tyrosine metabolism pathways annotated on KEGG, full pathways for the species were downloaded and the corresponding NCBI gene, nucleotide, and protein IDs were cataloged (Sayers et al. 2022). Protein domains were confirmed using HMMER v3.4 (Eddy 2008, 2009, 2011). Modules assigned in KEGG's tyrosine metabolism pathway were also identified. A full list of all species and their KEGG tyrosine metabolism pathways are listed in Supplementary material 1.

In this paper, we are referring to the melanin synthesis as depicted by the KEGG pathway tyrosine metabolism [KEGG:00350]. We separated out KEGG tyrosine metabolism pathway into four modules. Three of these modules are fully defined by KEGG: Catecholamine biosynthesis [M00042], Thyroid hormone biosynthesis [M00043], and Tyrosine degradation [M00044]. The fourth module, melanin synthesis, we have synthesized/adapted from KEGG and additional publications on tyrosine metabolism (D'Mello et al. 2016; Liu et al. 2016; Whitten and Coates 2017). This pathway includes multiple PO-candidates, for which this paper only refers to one group of PO-candidates: type-3 copper oxidases (prophenol oxidases, hemocyanins, and TYRs).

### Evolutionary relationships of PO-candidates

Evolutionary analyses of type-three copper oxidase proteins (prophenol oxidases, hemocyanins, and TYRs), were conducted in MEGA X (Kumar et al. 2018; Stecher et al. 2020). Nucleotide sequences were aligned with ClustalW's (Thompson et al. 1994) default parameters within the MEGAX system. The evolutionary history was inferred by using the neighbor-joining method (Saitou and Nei 1987), the associated taxa were clustered using a bootstrap test (1000 replicates), and evolutionary distance was computed using Poisson correction method (Zuckerkandl and Pauling 1965; Felsenstein 1985). The tree is drawn to scale, with branch lengths measured in the number of substitutions per site. Sequences used to create this tree can be found in Supplementary material 4.

### SCTLD experiment

The SCTLD transmission experiment used in this study was carried out at the University of the Virgin Islands (UVI) in April 2019, and its detailed experimental design and results are published in Meiling et al. (2021). The subsequent transcriptomic dataset and experimental analysis was published in Beavers et al. (2023). Briefly, this experiment obtained fragments from five species of stony coral (*Colpophyllia natans, Montastraea cavernosa, Orbicella annularis, Porites astreoides*, and *Pseudodiploria strigosa*) from the reef and split them into two smaller fragments. One fragment was placed into a control mesocosm equidistant from a central healthy *Diploria labyinthiformis* donor coral colony, while the corresponding fragment was placed into an experimental mesocosm in the same manner as the control, but with a SCTLD-infected *D. labyrinthiformis*. A total of eight genets of healthy and eight SCTLD infected *D. labyrinthiformis* were used for this paired design. In this study, corals in the mesocosm with SCTLD-infected *D. labyrinthiformis* were categorized during the eight-day experiment based on the phenotype of SCTLD appearing on the sample. If a coral obtained a lesion during the study, they were removed from the mesocosm as soon as there was 30% tissue loss. If a coral did not obtain a lesion during the eight-day experiment, it was classified as exposed to SCTLD. All corals in this study were flash-frozen and stored at –80°C for RNA sequencing. After the experiment, relative risk was calculated to represent a species risk of developing a lesion after exposure to SCTLD with each species having a unique relative risk associated with it. The relative risk also represents the species’ susceptibility to SCTLD, with increased relative risk values associated with high susceptibility to SCTLD. This study provides an experimental understanding of SCTLD in a diverse group of stony corals that vary in their evolutionary divergence and their known susceptibility to this particular disease, which makes it advantageous for the study of melanin as an immune response in stony corals. The full relevant phenotypes of the experiment such as disease status and relative risk can be found in Supplementary material 2.

### Melanin concentration extraction of SCTLD experiment samples

A Paasche airbrush filled with a buffer solution (50 mM TRIS pH 7.8 with 0.05 mM dithiothreitol) was used to remove coral tissue from the skeleton. Tissue slurry was homogenized, and cells were lysed using a PowerGen 125 tissue homogenizer with a medium saw tooth generator (Fisher Scientific). 1 mL of homogenate was flash-frozen and reserved for melanin analysis. Melanin protein analysis was used as a physical phenotype to represent the product for tyrosine metabolism.

The portion of extract reserved for melanin analysis was vacuum-dried using a Savant AES 1000 Automatic Environmental SpeedVac overnight. Following water evaporation, the total dry tissue weight in the 1-mL aliquot was determined. Then, tissue was disrupted by vortexing with a spatula of glass beads (∼200 µL in volume). Next, 300 µL of 10 M NaOH was added and the tubes were vortexed for another 30 s. To extract melanin, tubes were incubated for 48 h at room temperature in the dark. Following incubation, the tubes were centrifuged for 12 min at room temperature at 1500 RPM. The supernatant (40 µL) was transferred to two replicate ½ well UV plates. Absorbance was recorded at 410 nm and a standard curve of melanin dissolved in 10 M NaOH was used to convert absorbance to mg melanin (Mydlarz and Palmer 2011; Fuess et al. 2016). Replicate melanin concentration values were averaged across the two plates. Results are presented as µg of melanin per mg of tissue. Final melanin concentrations from all disease states and species were then analyzed for significance using a Kruskal–Wallis test (Kruskal and Wallis 1952) to compare melanin concentration to species and diseases status. A Dunn (1964) Kruskal–Wallis multiple comparison was used to compare between the five species and three diseases statues (Dinno 2015). *P*-values were adjusted with the Holm method.

### Expression of tyrosine metabolism in SCTLD infected corals

In the transcriptomic experiment of the SCTLD exposure study, samples from all five species were sequenced as detailed in Beavers et al. (2023), including the orthologous gene count matrix. Briefly, the count matrix was generated by identifying orthologous genes from a program called Orthofinder, and then normalized accordingly to the experiment. Orthofinder, a program used to provide phylogenetic inferences of orthologs, was used to identify groups of orthologous genes across predicted proteomes of all five coral species, which is referred to as “orthogroups” or “orthologs” (Emms and Kelly 2015; Traylor-Knowles et al. 2021). The orthogroups count matrices were generated for each species using Tximport (Soneson et al. 2016), and then merged based on orthogroups ID across all five coral species. The counts were then rlog normalized in DESeq2 (Love et al. 2015) using the design ∼species + treatment, removing orthogroups that had a rlog expression average of <10. For this study, the comparison of the rlog-transformed expression of the orthologs across all the coral species for the purpose of finding orthologous genes across divergent stony corals to create a cohesive understanding of genes affiliated with melanin production. The full experimental results for this transcriptomic experimental study are presented in Beavers et al. (2023) and the orthologous gene expression can be found in Supplementary material 2.

To identify orthologous genes correlated to melanin concentration, orthologous gene expression from Beavers et. al (2023)’s SCTLD exposure study was obtained and filtered for tyrosine metabolism pathway genes. After normalization, the human tyrosine metabolism pathway in KEGG was used as a reference to identify orthologs annotating as being associated with melanin synthesis. To confirm annotation of these orthogroups was correct, the corresponding protein sequences were searched for Pfam domains using HMMER (Eddy 2008, 2009, 2011), with a threshold of e-value <0.01. Coral orthogroup domain composition was then compared to human, and orthogroups lacking catalytic domains were excluded from further analysis. Orthogroups that passed this filtering were then correlated to melanin concentration using Pearson correlation analysis. If there were multiple orthogroups that were both correlated to melanin production and contained the same catalytic domains, a representative orthogroup was selected by determining overall pattern of correlation (majority negative or majority positive) of all orthogroups with the catalytic domains, and the orthogroup with the highest R-value was selected as the representative.

### Modules of tyrosine metabolism pathway annotation in stony corals

KEGG defines pathway modules as a molecular pathway within tyrosine metabolism, which include thyroid hormone biosynthesis, catecholamine biosynthesis, and tyrosine degradation. In this study, we adapt the KEGG tyrosine metabolism pathway to include melanin synthesis, which is included in the tyrosine metabolism pathway but not assigned a module number. Orthogroups with putative roles in melanin synthesis and correlations to melanin concentration were overlayed onto the adapted tyrosine metabolism pathway. This presence of orthogroups and correlation direction to melanin concentration was used to determine completeness of stony coral melanin synthesis pathway based on the evolutionary similarities and potential investments in cnidarian species compared to the human counterpart. All genes are available in Supplementary material 3.

## Results

### Evolutionary relationships of PO-candidates

Evolutionary analysis of type-three copper oxidase PO-candidates was conducted in MEGAX (Kumar et al. 2018; Stecher et al. 2020), which resulted in a phylogenetic tree that describes their evolutionary relationships. The optimal tree had a sum of branch length = 11.0307. The percentage of replicate trees in which the associated taxa clustered together in the bootstrap test (1000 replicates) are shown next to the branches (Felsenstein 1985). The evolutionary distances were computed using the Poisson correction method (Zuckerkandl and Pauling 1965) and are in the units of the number of amino acid substitutions per site. This analysis involved 18 amino acid sequences. All ambiguous positions were removed for each sequence pair (pairwise deletion option). There was a total of 965 positions in the final dataset. The identified Pfams domains for each of the sequences are displayed next to their evolutionary tree brand and each domain's color is coordinated based on Pfam domain e-value associated with each sequence if multiple domains were significant to a sequence.

**Fig. 1:**
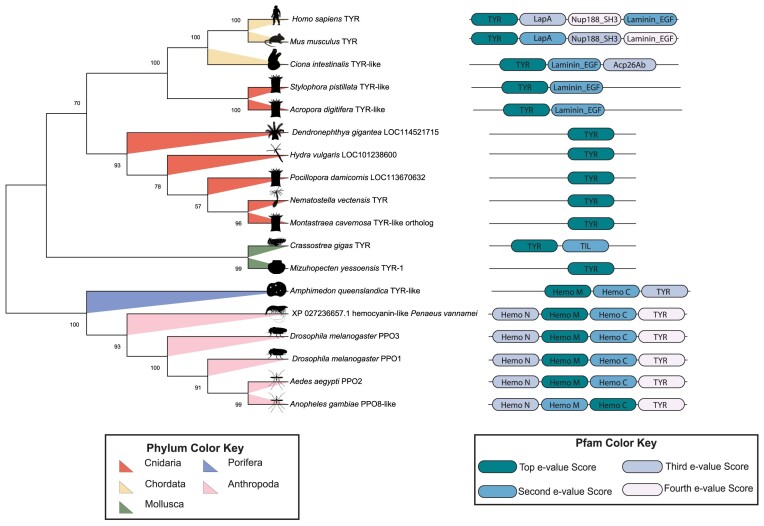
Neighbor-joining tree of type-three copper oxidase proteins. The phylogenetic tree depicted was created in MEGAX using the neighbor-joining method. The tree has a sum branch of 11.0307. The percentage of replicate trees in which the associated taxa clustered together are shown next to the branches. There was a total of 965 position in the final dataset. Phyla of each taxa are highlighted on the branches of the tree, showing branching based on phyla. Pfam domains significant in HMMER search for the PO-candidates. Domains include TYR (PF00264.24), LapA - LapA_dom (PF07974.17), Nup188_SH3 - Nup188_SH3-like (PF21094.1), Laminin_EGF (PF00053.28), Hemo_M - Hemocyanin_M (PF00372.23), Hemo_C - Hemocyanin_C (PF03723.18), Hemo_N - Hemocyanin_N (PF03722.18), and TIL (PF01826.21). If multiple Pfam domains were associated with one protein sequence, color assignment was based on top and subsequent Pfam domain hit in HMMER based on e-value per sequence.

There are two major branches of the tree generated (Fig. 1). The first major branch consists of representatives from phyla Chordata, Cnidaria, and Mollusca. The Chordata branch with two Cnidaria species; *Stylophora pistillata* and *Acropora digitifera*. Cnidarian species; *Dendronephthya gigantea, Hydra vulgaris, Pocillopora damicornis, Nematostella vectensis* and the predicted TYR orthogroup from *M. cavernosa* all group together. The molluscan species are an outgroup of this first branch. The second branch includes members from phyla Anthropoda and Porifera. The poriferan species *Amphimedon queenslandica* acts as an outgroup of the arthropodan species.

In the phylogenetic tree, we also see branching based on Pfam domain composition. There is a total of nine Pfams that were assigned at a significant e-value (<0.01) to the target sequences (Supplementary material 4). Chordate's all two of the same Pfam domains: TYR (PF00264.24) and Laminin_EGF (PF00053.28). Mammalian species *Mus musculus* and *H. sapiens* have additional two Pfam domains: LapA_dom (PF07974.17) and Nup188_SH3-like (PF21094.1). Cnidarians that groups with the chordates also had Pfam domain Laminin_EGF. The rest of the cnidarians only had Pfam domain TYR. In the Mollusca branch, both species have the TYR Pfam. Only *Crassostrea gigas* has Pfam domain TIL (PF01826.21). The second branch which consisted of the porifera and arthropods all have Pfam domains Hemocyanin_M (PF00372.23), Hemocyanin_C (PF03723.18), and TYR. All arthropods also have the Hemocyanin_N (PF03722.18) domain. Overall, species evolution within phyla and Pfam domain assignment drive the phylogenetic tree.

### Cnidarian melanin synthesis in active disease dataset

**Fig. 2:**
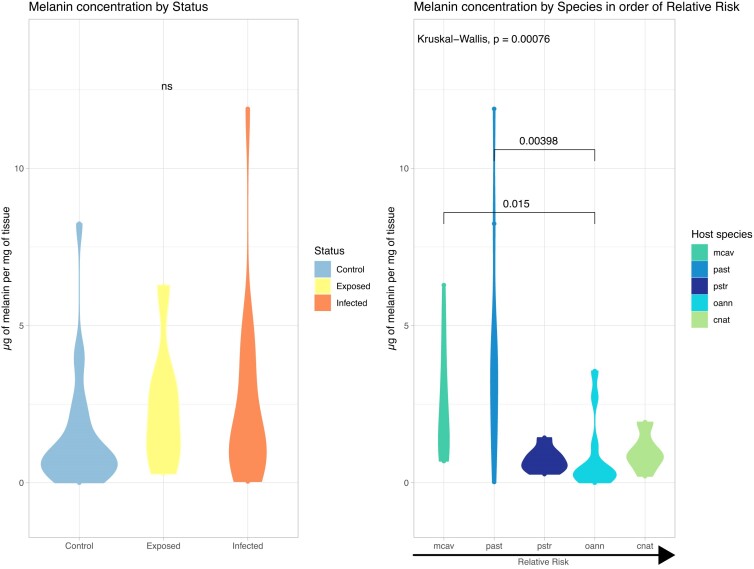
Melanin concentration by disease status and species. In this study, Kruskal–Wallis test was used to test melanin concentration differences based on disease status and species. There is no significant difference in melanin concentration between status, but there is significant difference between species (chi-squared = 19.061, df = 4, *P*-value = 0.0007647). Dunn (1964) test on Kruskal–Wallis multiple comparison *P*-values adjusted with the Holm method are available in Tables 1 and 2. Overall concentration of melanin by species had an inverse relationship to relative risk, with low-susceptible species such as *M. cavernosa* having a higher concentration of melanin while high-susceptible species such as *O. annularis* had lower concentrations of melanin.

While melanin concentration did not significantly change between disease states (Kruskal–Wallis chi-squared = 4.1406, df = 2, *P*-value = 0.1261, Table 1), there was significant difference in between species (Kruskal–Wallis chi-squared = 19.061, df = 4, *P*-value = 0.0007647, Table 2), and their associated relative risk, as seen in Fig. 2. There was an inverse relationship of relative risk of disease and melanin concentration. Species that are highly susceptible to SCTLD, like *O. annularis*, had significantly lower melanin concentration compared to more disease resistant species like *M. cavernosa* and *P. asteroides*

**Table 1:** Dunn (1964) Kruskal–Wallis multiple comparison *P*-values with the Holm method melanin concentration to disease status.

Comparison	Z	P.unadj	P.adj
Control - exposed	−1.574376	0.11540060	0.2308012
Control - infected	−1.689263	0.09116895	0.2735069
Exposed - infected	0.430470	0.66685377	0.6668538

This table depicts the Dunn (1964) test on the Kruskal–Wallis multiple comparison of melanin concentration to disease status using a *P*-value adjustment with the Holm method. In this study, no comparison has a significant difference in melanin concentration, demonstrating that no difference in melanin concentration based on SCTLD exposure or infection.

**Table 2:** Dunn (1964) Kruskal–Wallis multiple comparison *P*-values with the Holm method melanin concentration to species.

Comparison	Z	P.unadj	P.adj
cnat - mcav	−1.9172247	0.0552093949	0.276046974
cnat - oann	1.1232832	0.2613172238	1.000000000
mcav - oann	3.1423822	0.0016757916	0.015082124
cnat - past	−2.3622522	0.0181642821	0.127149975
mcav - past	−0.5500672	0.5822732513	1.000000000
oann - past	−3.5411254	0.0003984242	0.003984242
cnat - pstr	0.5747049	0.5654908976	1.000000000
mcav - pstr	2.3215646	0.0202563896	0.121538337
oann - pstr	−0.4293336	0.6676804964	0.667680496
past - pstr	2.7184138	0.0065595745	0.052476596

This table depicts the Dunn (1964) test on the Kruskal–Wallis multiple comparison of melanin concentration to species using a *P*-value adjustment with the Holm method. In this study, there was significant difference in melanin concentration in species. The comparisons with significant difference after p-adjustment were Mcav-Oann and Oann-Past.

**Fig. 3:**
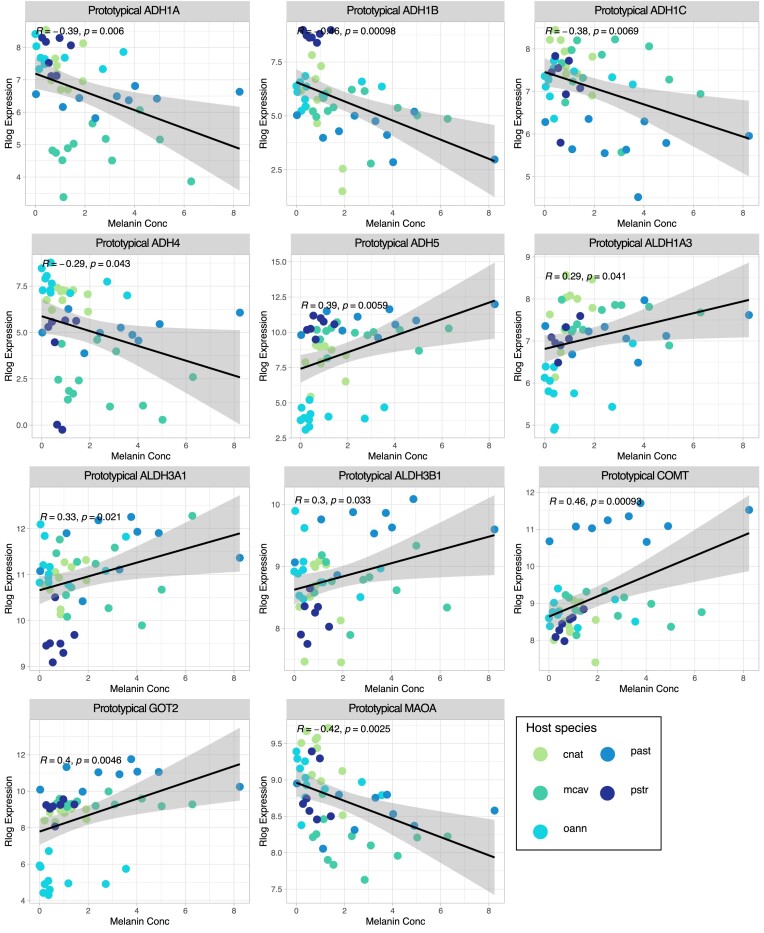
Tyrosine metabolism genes unassigned to a module Pearson correlation plots in this figure, Pearson correlation of rlog expression of 11 tyrosine metabolism genes to melanin concentration are visualized. Melanin concentration is established on the *x*-axis, and rlog expression on the y-axis. The five stony coral species cnat (*C. natan*), mcav (*M. cavernosa*), oann (*O. annularis*), past (*P. asteroides*), and pstr (*P. strigosa*) are colored on the graphs. All genes have a positive correlation (increased expression of gene with increased melanin concentration) except ADH1A, ADH1B, ADH1C, ADH4, and MAOA with negative correlation (decreased melanin concentration with decreased rlog expression).

A total of 313 orthogroups were identified by HMMER as putative tyrosine metabolism orthogroups in the five stony coral species. Out of these 313 orthogroups, a total of 109 orthogroups significantly correlated to melanin concentration (*P*-value < 0.05), that represent 19 genes from the tyrosine metabolism pathway. Furthermore, out of these 19 genes, 11 genes are not assigned to a KEGG module (Fig. 3, Table 3). The 11 genes not assigned to the KEGG module were split between positive and negative correlation.

**Table 3:** Non-module membered tyrosine metabolism pathway genes with significant Pearson correlation to melanin concentration.

Gene Name	Gene description	Orthogroup	Pearson correlation	*P*-value
ADH1A	Alcohol dehydrogenase 1A (class I), alpha polypeptide	OG0004227	−0.3868996	0.00602846
ADH1B	Alcohol dehydrogenase 1B (class I), beta	OG0003914	−0.4565233	0.00097698
ADH1C	Alcohol dehydrogenase 1C (class I), gamma polypeptide	OG0005371	−0.3808345	0.0069426
ADH4	Alcohol dehydrogenase 4 (class II), pi polypeptide	OG0010654	−0.2903729	0.04297246
ADH5	Alcohol dehydrogenase 5 (class III), chi	OG0007001	0.3875348	0.00593908
ALDH1A3	Aldehyde dehydrogenase 1 family member A3	OG0004406	0.29366773	0.0405594
ALDH3A1	Aldehyde dehydrogenase 3 family member A1	OG0002905	0.32896483	0.02100162
ALDH3B1	Aldehyde dehydrogenase 3 family member B1	OG0008949	0.30488644	0.03316219
COMT	Catechol-O-methyltransferase	OG0001002	0.45817774	0.00093114
GOT2	Glutamic-oxaloacetic transaminase 2	OG0002600	0.39805816	0.00461761
MAOA	Monoamine oxidase A	OG0004777	−0.42236	0.00250248

In this table, genes part of the tyrosine metabolism pathway that were not associated with a module within the pathway were identified as correlated to melanin concentration. The gene name, gene description, the corresponding orthogroup number, and the Pearson correlation and *P*-value (*P* < 0.05) are reported.

**Fig. 4:**
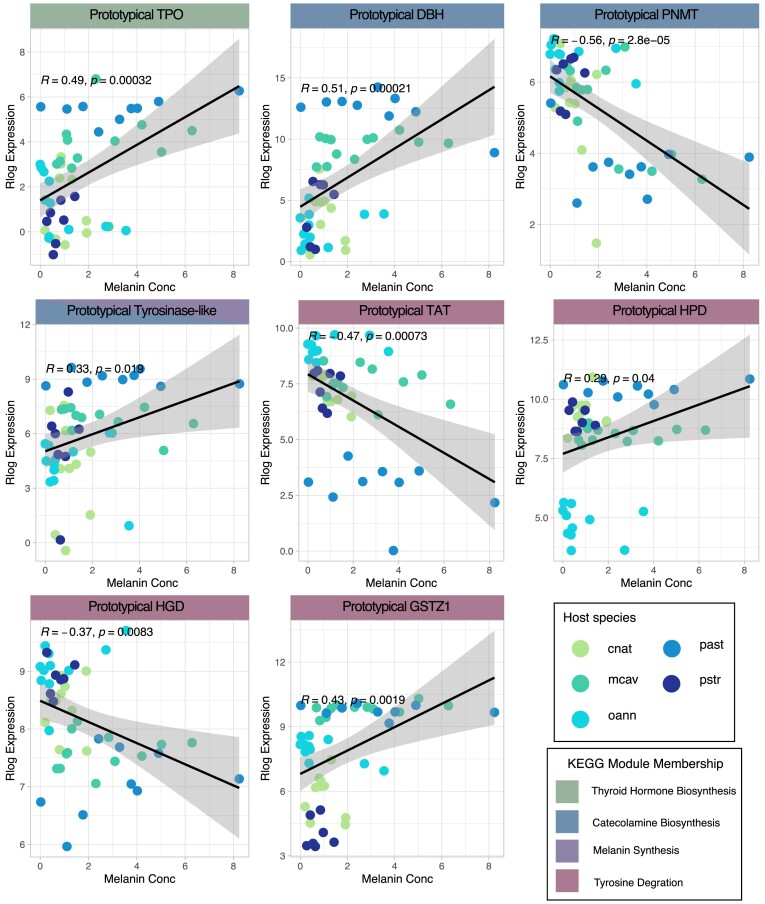
Tyrosine metabolism module membered genes Pearson correlation plots. In this figure, Pearson correlation of rlog expression of eight representative tyrosine metabolism genes to melanin concentration are visualized. Genes are categorized into KEGG module membership (Thyroid Hormone Biosynthesis, Catecholamine Biosynthesis, Melanin Synthesis, and Tyrosine degradation). Melanin concentration is established on the *x*-axis, and rlog expression on the y-axis. The five stony coral species cnat (*C. natan*), mcav (*M. cavernosa*), oann (*O. annularis*), past (*P. asteroides*), and pstr (*P. strigosa*) are colored on the graphs. Genes in modules have a mixed positive and negative correlation relationship to melanin concentration. Positive correlations include TPO, DBH, TYR-like, HPD, and GSTZ1. Negative correlations include PNMT, TAT, and HGD.

The remaining eight orthogroups were assigned to the four different modules depicted in Fig. 4 and Table 4: thyroid hormone biosynthesis, catecholamine biosynthesis, melanin synthesis, and tyrosine degradation. There is one assigned coral orthogroup, thyroid peroxidase (TPO), in the thyroid hormone biosynthesis module, which is positively correlated to melanin concentration. In the catecholamine biosynthesis pathway, all five putative orthogroups were found in the stony corals. Three of the five orthogroups correlated to melanin concentration, with TYR and phenylethanolamine N-methyltransferase (PNMT) having a negatively correlation and dopamine beta-hydroxylase (DBH) having a positive correlated to melanin concentration. Orthogroup OG0002134 was found to be a significant gene in the melanin synthesis module. In the human pathway, there are three TYR-like proteins: tyrosine-related-protein 1 (TYRP1), dopachrome tautomerase (DCT), and TYR. Orthogroup OG0002134 matched to these three TYR-like proteins and was positively correlated to melanin concentration. For this module, we consider orthogroup OG0002134 to be a TYR-like protein (TYR-like), as TYRP1, DCT, and TYR all have functional Pfam domain TYR in humans. However, there is a unique domain associated with DCT, which eliminates this gene from being an orthogroup found in this study. In the tyrosine degradation module, four of the five coral orthogroups are correlated to melanin concentration with 4-hydroxyphenylpyruvate dioxygenase (HPD) and glutathione S-transferase zeta 1 (GSZT1) are positively correlated to melanin concentration and tyrosine aminotransferase (TAT) and homogentisate 1,2-dioxygenase (HGD) are negatively correlated to melanin concentration. Ultimately, the modules defined by tyrosine metabolism are mixed when it comes to correlation to the melanin product.

**Table 4:** Module membered tyrosine metabolism pathway genes with significant Pearson correlation to melanin concentration.

Gene name	Gene description	Orthogroup	Pearson correlation	*P*-value	Module membership
DBH	Dopamine beta-hydroxylase	OG0000903	0.50545755	0.00021183	Catecholamine biosynthesis
GSTZ1	Glutathione S-transferase zeta 1	OG0000629	0.43246004	0.00191403	Tyrosine degradation
HGD	Homogentisate 1,2-dioxygenase	OG0008694	−0.3731272	0.00827644	Tyrosine degradation
HPD	4-hydroxyphenylpyruvate dioxygenase	OG0012367	0.29395785	0.04035235	Tyrosine degradation
PNMT	Phenylethanolamine N-methyltransferase	OG0010759	−0.5605623	2.80E-05	Catecholamine biosynthesis
TAT	Tyrosine aminotransferase	OG0001485	−0.4665614	0.00072717	Tyrosine degradation
TPO	Thyroid peroxidase	OG0004407	0.49266186	0.00032306	Thyroid hormone biosynthesis
TYR-like	Tyrosinase-like	OG0002314	0.33284132	0.01944932	Melanin synthesis and catecholamine biosynthesis

In this table, genes part of the tyrosine metabolism pathway that were associated with a module within the pathway were identified as correlated to melanin concentration. Gene name, gene description, the corresponding orthogroup number, and the correlation and *P*-value (*P* < 0.05), and associated module are reported.

## Discussion

**Fig. 5:**
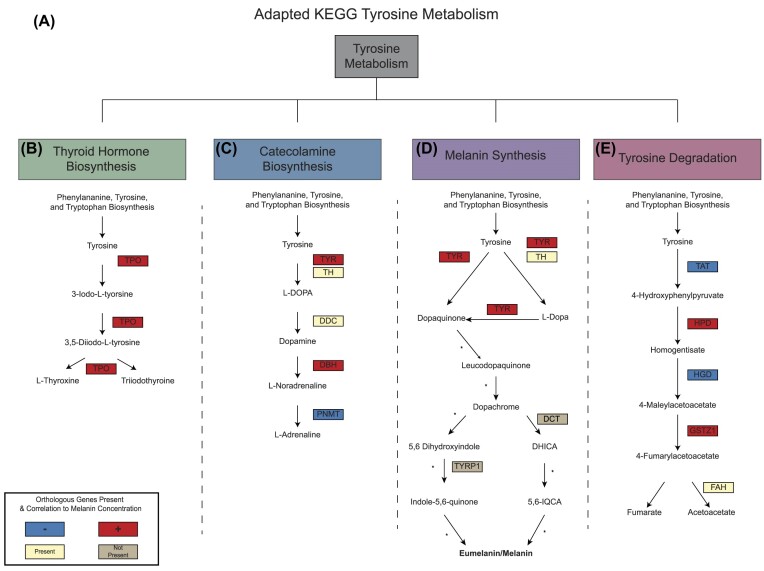
Adapted tyrosine metabolism pathway in stony corals. The tyrosine metabolism was adapted from KEGG from *H. sapiens*, the species with the closest evolutionary relationship to the PO-candidates found in cnidarians. Overlayed are the orthogroups correlation to melanin concentration (bolded as eumelanin/melanin on the tyrosine metabolism adapted pathway). Orthogroups are colored based on their correlation to melanin concentration; positive (red), negative (blue), no correlation but present in the orthologs genes (yellow), not present in stony coral (gray). TH, DDC, and FAH were present as orthologous genes in the stony coral expression but were not correlated to melanin concentration. TYRP1 and DCT were considered not found in the orthogroups as they have uniquely evolved in humans.

This study provides a comprehensive analysis of the genes involved in tyrosine-mediated melanin synthesis in cnidarians. The study met its first goal by generating a phylogenetic tree that explained the phylogenetic relationship of protein family type-three copper oxidase proteins. The second goal resulted in putative stony coral genes being correlated to melanin concentration and the identification of an adaptive tyrosine metabolism pathway in stony corals. We have several key findings: cnidarians have TYR protein domains and group with humans while PO-candidates with hemocyanin domains exist mainly in insect groups, stony corals have correlated expression of melanin product to TYR in a disease susceptibility context, and stony coral have an evolutionary conserved tyrosine metabolism pathway (Fig. 5).

### Evolutionary relationships of TYR

The structural and evolutionary analysis of PO-candidates across 18 species indicates a divide of type-three copper oxidase proteins based on protein domains. Interestingly, cnidarians PO-candidates consistently group with evolutionary distant *H. sapiens* compared to more closely related arthropod species. Many coral immune genes are also similar to the human genes that are important for human system development, cell signaling, or immune response (Miller et al. 2005; Watanabe et al. 2009; Steele et al. 2011; Mansfield et al. 2017; Williams and Gilmore 2020). The PO-candidates involved in melanin synthesis may reflect another conserved history between humans and cnidarians. While this study found all PO-candidates contain a TYR Pfam domain, the exclusivity of TYR domains in cnidarians and chordates and the lack of hemocyanin domains may be driving this similarity. The principles of phylogenetic studies and evolutionary processes that drive biological diversification are relevant at both gene and species levels (Herrada et al. 2011). From a protein family perspective, the type-three copper oxidase evolutionary studies may support TYR being a conserved functional domain that evolved from basal metazoans. Evolutionary studies of the diversity of type-three copper proteins have indicated two ancient gene duplication events, as well as differential loss and expansions within specific phyla that could support the evolutionary relationships identified in this study (Aguilera et al. 2013). After these duplication events and differential loss and expansions, arthropods may have developed derived traits associated with their specificity in PO proteins that drive the evolutionary distance between cnidarians and arthropods (Wang et al. 2017, 2018). From a species perspective, the unique evolutionary history of insects may continue to separate them from humans and cnidarians. Insects seem to have an extensive investment in melanin as a primary immune response as evident from their highly specialized PO-candidates that can be phenotypically unique to specific pathogens or specific immune responses (Binggeli et al. 2014; Dudzic et al. 2015). This not only makes comparison to cnidarians and humans difficult but also identifies a key evolutionary event that could be driving these relationships identified in this study. Overall the type-three copper oxidase protein family is heavily influenced by the protein domains assigned to each species.

The characterization of coral PO-candidates has remained elusive due to the implications of multiple PO-candidates in previous coral melanin precursor studies (Mydlarz and Palmer 2011). There is the possibility of a laccase being a contender for the PO-candidate that can characterize coral melanization, as laccase has been found in coral gene expression and proteomic datasets (Palmer et al. 2012; Vidal-Dupiol et al. 2014; Ricci et al. 2019; Connelly et al. 2020), and is a conserved multicopper oxidase in all animals (Janusz et al. 2020). This gene, however, is not annotated to KEGG's tyrosine metabolism pathway and most likely will remain that way until clarity on laccase's role in melanin synthesis is found (Hansakon et al. 2020; Bajpai et al. 2023). Increasing the number of identified and annotated genes for laccases in the KEGG annotation of tyrosine metabolism, as well as increasing the limited number of annotated PO-candidates, will provide resolution for laccases role in melanin production. However, this does not negate the very important role that TYR plays in cnidarians.

TYR is supported as the primary PO-candidate in coral immunity as it's associated with immune cells identified in stony corals. Single-cell sequencing found a putative immune cell type that was enriched with TYR (Levy et al. 2021). As such, this cell was assigned as one of only two immune cells in corals, with further functional studies confirming the presence of two distinct types of immune cells in cnidarian species (Snyder et al. 2021). These cells bare a similarity to *H. sapiens* melanocytes, which produce melanin (Gasque and Jaffar-Bandjee 2015) due to their unique and specialized proteins, functionality, and their location in the epithelial layer (Palmer et al. 2011; Fuess et al. 2018; Ricci et al. 2019). In addition, histological studies and visualization have implicated melanin barriers in the mesoglea that is where the putative immune cells primarily reside (Vargas-Ángel et al. 2007; Tracy et al. 2021). The presence of TYR in relation to SCTLD susceptibility also offers a unique perspective on how immune cell types may be utilized for preventive immune responses in cnidarians (Netea et al. 2019; Prigot-Maurice et al. 2022). It could be hypothesized that TYR association with an immune cell type may employ melanin product as a preventive measure at the beginning of a disease event to mitigate the infection. In conclusion, the evolutionary conserved presence of TYR as a PO-candidate leads to the understanding of how immune response occurs within cnidarian species based on the cell types it is originating from.

### Tyrosine metabolism genes and melanin concentration reveals immune responses and homeostasis mechanisms in stony corals

The tyrosine metabolism pathway is divided into four modules: (i) thyroid hormone biosynthesis, (ii) catecholamine biosynthesis, (iii) melanin synthesis, and (iv) tyrosine degradation. Modules are identified based on the ending products, such as tyrosine to triiodothyronine/thyroxine in the thyroid hormone biosynthesis module, tyrosine to adrenaline in the catecholamine biosynthesis module, tyrosine to melanin product in melanin synthesis, and tyrosine to a homogentisate in tyrosine degradation. Using these pathways, gene expression from five stony corals in an experimental SCTLD exposure study was leveraged to look for associations between the genes within these modules and melanin concentration.

Melanin concentration did not vary between disease states, with no apparent upregulation of melanin concentration in response to SCTLD, suggesting that melanin is not the primary immune response in this disease. Melanin has been documented as a primary immune response in other coral diseases, such as Sea fan—aspergillosis system and Eunicea Black Band Disease (Mydlarz and Harvell 2007; Fuess et al. 2018; Ricci et al. 2019). While the causative agent of SCTLD is unknown, it appears that the algal symbiont is afflicted during infection and other host immune responses are initiated (Landsberg et al. 2020; Beavers et al. 2023). However, the importance of melanin in SCTLD immune response may be in preventing disease signs in certain coral species.

Melanin concentration varied significantly between species within the study, suggesting a constitutive role of melanin in immunity to SCTLD. The melanin concentration inversely related to relative risk or susceptibility to SCTLD, with more resistant species having higher overall concentrations of melanin while more susceptible species had overall lower melanin concentrations, indicating the potential role melanin plays as resistance immune trait. The PO-candidate TYR has previously been found as a resistance train in another tissue loss disease, white plague, with the gene being identified as lineage specific, a gene that varied by species but not by disease state (MacKnight et al. 2022). This supports the important role melanin may play in the prevention of lesions in stony corals by acting in a resistance role.

Even though melanin concentration was not significant in disease states, we can still use the phenotypic data of melanin concentration to help contextualize the gene expression data. By correlating coral gene expression data to melanin concentration, we can identify key genes and pathways relevant to melanin production in stony corals. Most importantly, this study identified orthologous genes of the five cnidarian species that were found to have Pfam domain matching the *H. sapiens* tyrosine metabolism pathway genes, grounding the method of comparative immunology of stony corals to humans.

The unassigned tyrosine metabolism and positive thyroid hormone biosynthesis putative orthogroups may play a role in oxidative stress in stony corals. Melanin synthesis increases the risk oxidative stress in tissues for two major reasons: (i) the pro-oxidant state in melanin synthesis, and (ii) the antioxidant state that normally occurs with pathological conditions when melanin synthesis is activated (Denat et al. 2014). The majority of the unassigned putative orthogroups are alcohol dehydrogenases and other amine neurotransmitters. Alcohol dehydrogenases have been associated with reducing injury during a disease, such as mitigating liver damage (Hou et al. 2019), and have a positive correlation in melanin product in corals, indicating a similar mitigation role. In addition to this, amine neurotransmitter MAOA in this study had a negative correlation to melanin concentration. Increased MAOA expression has been correlated to the development of pigmentation disorders (Enkhtaivan and Lee 2021). The negative correlation of MAOA to melanin in corals could be mitigating the adverse effects of melanin production in cnidarians. The thyroid hormone biosynthesis module only has one enzyme; TPO, which is an enzyme involved in peroxidase activity and response to oxidative stress in a variety of vertebrate and invertebrates (Taurog 1999). While traditionally associated with thyroid hormone synthesis in humans (Ruf and Carayon 2006), the protein itself has peroxidase enzymatic capability that is important to oxidative stress. Peroxidase activity has been shown to be an important immune response in corals during a variety of stressors such as infections, injury, or heat stress (Mydlarz and Harvell 2007; van de Water et al. 2015; Fuess et al. 2016). The relationship of TPO being positive to melanin concentration in this study indicates an ability to response to oxidative stress using this specific pathway. In addition, TPO's correlation to melanin product indicates a potential dual function, the role of tyrosine metabolism and the ability to perform antioxidant activity.

The stony coral catecholamine biosynthesis module has a putative orthogroups positively and negatively correlated to melanin concentration. Catecholamine is associated with neurotransmission, as it is classified as a slow neurotransmitter. Slow transmitters and other neurologically relevant products such as adrenaline have been identified in cnidarian species in some capacity, but mechanisms for their production remain elusive (Kass-Simon and Pierobon 2007). Currently, it is indicated that cnidarian genomes do not contain the specific rate-limiting enzymes involved in catecholamine biosynthesis based on comparative approaches, and instead many have unique cnidaria specific enzymes present to perform this pathway (Moroz et al. 2021). In this study, we have found orthologous genes of five stony corals that have Pfam domains that may function in catecholamine biosynthesis. This can be used in future comparative studies to identify neurotransmitter production in cnidarians.

In the search for the PO-candidates responsible for melanin synthesis in stony corals, this study found one of 14 TYR domain containing orthogroups to be correlated to melanin synthesis. This positive correlation indicates biological significance of this particular orthogroup as the amount of melanin product follows orthogroup expression. There were a total of 13 orthogroups with TYR Pfam domains that were not significantly correlated to melanin product, indicating a diversity of TYR-like genes in corals. It has been proposed that a diversity of TYR-like genes in cnidarian species may indicate an organism's ability to launch either specific responses or stronger responses to pathogen challenges and promoting disease resistance (Bailey et al. 2019), and multiple copies have been found in *Exaiptasia pallida* and *N. vectensis* and *Porites australiensis* (Anctil 2009; Bailey et al. 2019; Shinzato et al. 2021). The lack of correlations to melanin in the SCTLD dataset may point to the utility and specificity of these orthogroups in other immune capacities. Similar to the expansion of other immune genes in cnidarians (Emery et al. 2021), the expansion of TYRs could contribute to greater immune specificity, especially to overcome the lack of an adaptive immune system.

The number of cnidarians orthogroups may also reflect the expansion of TYR-like proteins similar to human TYR-like protein expansions. In humans, there have been multiple evolutionary events in TYRs that resulted in DCT and other TYR-related-proteins (TYRPs1) within melanin synthesis pathways (Budd and Jackson 1995; Sturm et al. 1995; Camacho-Hübner et al. 2002) and have specific functions and implications in disease pathogenesis. While it is possible that stony corals could use TYR-like proteins, it is not expected that the orthogroups found is a TYR related protein or DCT gene due to this evolutionary event. However, the identification of multiple orthogroups that have TYR domains with only one orthogroup having significant to melanin concentration in this exposure study provides support to the theory of the expansion of immune genes in cnidarians for greater immune specificity.

The identification and expression of all putative orthogroups in the tyrosine degradation module in the stony coral SCTLD dataset, indicates an evolutionary-conserved investment in this pathway. Basal metazoan, like the stony corals in this study, have an open-body plan, which is susceptible to disruptions in cellular homeostasis. While melanin synthesis can be an important and necessary component of the immune response, tyrosine isomer accumulation during tyrosine metabolism has a pathological association with free radicals that lead to oxidative stress and inflammation (Molnár et al. 2016; Ipson et al. 2019), disrupting cellular homeostasis. By degrading tyrosine from the cell, an organism can effectively defend against oxidative stress associated with melanin synthesis (Nguyen et al. 2020). Oxidative stress leads to tissue damage (Palmer et al. 2011), and the dysbiosis of algal symbionts (Weis 2019), and is critical to homeostasis of a cnidarian cell. The positive correlation of genes HPD and GSTZ1: This module indicates the homeostatic maintenance for the stony coral's open body plan. Humans, with melanocytes, have multiple pathways and regulations set up to avoid ROS stress within melanocytes and can keep the stress localized (Denat et al. 2014). Cnidarians may rely on several pathways within their immune response to actively decrease tyrosine buildup to avoid tissue damage due to unregulated ROS stress. The cnidarian tyrosine degradation module is likely highly conserved due to the same reasons. Overall, the identification of putative orthogroups in stony corals provides targets for further understanding the tyrosine metabolism pathway in the context of disease and provides the first look at genes involved in melanin production and its role in disease response.

## Conclusions

Understanding cnidarian tyrosine metabolism pathway can help cognize the evolutionary events that influenced melanin synthesis pathway and the mechanisms they use to maintain cellular integrity. In this study, cnidarian and coral melanin synthesis are associated with PO-candidate TYR, grouping with chordates such as humans in phylogenetic studies due to protein domain assignment. TYR was also found to have multiple copies in stony corals. The pattern of human similarity to human elements of immunity hold true for PO-candidates, as seen in other immune functions in cnidarians. Pattern recognition receptors such as nod-like receptors and toll-like receptors are more similar to human genes than other invertebrates and even have expansion in these receptors such as domain combinations not found within their human counterparts (Dimos et al. 2019; Emery et al. 2021). The combination of these studies with our study, we demonstrate that downstream immune cascades follow this pattern of evolutionary similarity and increased repertoire of functionality as in cnidarian receptors.

It is now more important than ever to identify key pathways in cnidarian immune systems as coral disease is a rising threat to reef ecosystems. This study provides 18 putative orthogroups from five stony corals that define tyrosine metabolism, a pathway involved in melanin synthesis, oxidative stress response, and tyrosine degradation as evidence by the correlation of orthologous gene expression to melanin concentration. Melanin is identified in this study as a potential immune resistance train in tissue loss diseases that helps to understand how stony corals may fight pathogens. The methods of this paper provide a blueprint for future comparative studies to obtain other biologically important cnidarian immune pathways.

## Author contributions

E.V.B. and I.P. conceived of the project. Data collection was performed by E.V.B., I.P., K.M.B., A.S., and M.C. Bioinformatic analysis was conducted by E.V.B., I.P., A.S., M.C., and M.E. E.V.B., M.E., and L.M. wrote the manuscript. All authors contributed to the article and approved the submitted version.

## Supplementary Material

icae115_Supplemental_Files

## Data Availability

Data for this project are available in previous publications Meiling et al. (2021) and Beavers et al. (2023). The raw RNAseq data generated from Beavers et al. (2023) that were used in this project are deposited in the NCBI database under accession code PRJNA860922. Additional data are available in Supplementary materials.

## Notes

From the symposium “Immunity in the ‘omics age: what can ‘omics approaches tell us about immunity in natural systems?” presented at the annual meeting of the Society for Integrative and Comparative Biology, January 16–March 31, 2024.
